# Young Vs Adults: Adaptability to ICTs, resilience, anxiety and depression in university students and professors

**DOI:** 10.1192/j.eurpsy.2023.990

**Published:** 2023-07-19

**Authors:** U. Rodriguez-De Avila, Z. Paba-Argote

**Affiliations:** Cognition and Education Research Group, Universidad del Magdalena, Santa Marta, Colombia

## Abstract

**Introduction:**

Personal access to digital technologies proved to be an important measure to curb the desertion of students at a public university in the Colombian Caribbean during the COVID-19 pandemic; however, there are no studies that explore the relationship between the use of new information and communication technologies, resilience, anxiety and depression during physical isolation derived from the public health situation, in this geographic area.

**Objectives:**

Analyze adaptability to the use of technologies and its relationship with resilience, anxiety and depression in university students and professors in the midst of isolation by Covid-19.

**Methods:**

The sample consisted of 328 subjects, aged between 18 and 69 years (30.6±12.21), 39% men and 61% women; 67.4% young students and 32.6% professors. The study was quantitative, exploratory, by convenience, online. The instruments were registered on the Web and were provided through WhatsApp, Facebook and personal mail by means of a Snowball type sample selection. It was developed during the period of mandatory physical isolation, decreed in the first quarter of 2020 by the Colombian State due to the COVID-19 Pandemic. The analysis was performed using descriptive, correlational and inferential statistics. The Kolmogorov-Smirnov (KS) normality test was applied, confirming a non-normal distribution of the sample. A correlational analysis was performed using Kendall’s Tau-b correlation coefficient and for the subsequent analysis of variance (segmented by age), Kruskal-Wallis Chi-square (X2) was used, verifying the variances by post hoc. In the case of the analysis of variance segmented by occupation (professors and students) and by sex, the Mann-Whitney U X2 test was used.

**Results:**

Of the total sample, 86.3% showed maladaptability to the use of ICTs, with no significant difference between professors and students (p=0.48). Resilience is higher in professors than in students (p<0.01); anxiety and depressive symptoms are higher in students (p<0.01). Adaptability was inversely associated with Resilience and directly with Anxiety and Depressive Symptoms (p<0.01); the highest risk group are students under 22 years old. A future publication will expand on the details of the results.

**Conclusions:**

It is concluded that maladaptability to the use of ICTs may be associated with contextual elements not studied in the present study, however, the mental impact remains high mainly in the younger student population, especially in times of general social crisis. Credit is given to the project BPIN 2020000100758: Development of an Integrated Technological System for the promotion of mental health, psychosocial and socioemotional problems and prevention of gender violence, caused by the COVID19 pandemic in the Magdalena region, which allowed the deepening for the analysis of the results. Likewise, to Universidad del Magdalena for its support in installed capacity.

**Disclosure of Interest:**

None Declared

